# Reproductive health at crossroads: progress and challenges since the International Conference on Population and Development in Cairo

**DOI:** 10.1007/s42379-025-00208-4

**Published:** 2026-01-09

**Authors:** Fan Yang, Heini Väisänen

**Affiliations:** 1 Institute of Population Research, Peking University, 5 Yiheyuan Rd, Haidian District, Beijing, China; 2 Institut National d’études Démographiques (INED), 9 Cours des Humanités, 93300 Aubervilliers, France

**Keywords:** Sexual and reproductive health and rights (SRHR), ICPD programme of action, Reproductive inequalities

## Abstract

Thirty years after the 1994 International Conference on Population and Development (ICPD) in Cairo, sexual and reproductive health and rights (SRHR) stand at a critical juncture. This commentary reviews the progress, persistent gaps, and new challenges since ICPD. It focuses on three main domains along the reproductive continuum: prevention and treatment of sexually transmitted infections (STIs), including HIV; infertility and assisted reproductive technologies; and pregnancies not ending in live births, including induced abortion, miscarriage, ectopic pregnancy, and stillbirth. Since ICPD in Cairo, scientific advances have transformed prevention and care. These advances have greatly improved population health and expanded reproductive options. However, their benefits are unevenly distributed. Socioeconomic inequities persist; marginalized populations are the least likely to benefit from innovation. Meanwhile, antimicrobial resistance, stigma, and underfunded health systems threaten sustainability. In infertility care, global disparities in access to services coexist with risks of over-medicalization and commercialization in some high-income settings. For pregnancies not ending in live births, restrictive abortion policies, ongoing stigma, and lack of recognition of miscarriage and stillbirth continue to undermine health, autonomy, and equity. Emerging pressures—including climate change, demographic shifts, and declining political and financial support to SRHR—put progress at risk. We argue that recommitment to the ICPD’s rights-based, human-centered framework is needed. The future SRHR agenda must combine scientific innovation with social justice, solid financing, ethical governance, and intersectional policy. Only then can we protect gains and prevent deeper reproductive inequalities.

## Introduction

Last year marked the 30th anniversary of the International Conference on Population and Development (ICPD) conference in Cairo, Egypt in 1994, which is often hailed as a landmark moment for the international recognition and definition of sexual and reproductive health and rights, which were situated within the larger framework of human rights (Becquet et al., 2024). The Programme of Action defines these rights as follows:

These rights rest on the recognition of the basic right of all couples and individuals to decide freely and responsibly the number, spacing and timing of their children and to have the information and means to do so, and the right to attain the highest standard of sexual and reproductive health. It also includes their right to make decisions concerning reproduction free of discrimination, coercion and violence, as expressed in human rights documents (UN Department of Economic & Social Affairs, 1994)

Since then, sexual and reproductive health and rights have made their way to other international policy agreements, such as Sustainable Development Goals (United Nations, 2015a), where a definition of reproductive health was also mentioned (Ann M Starrs et al., 2018a, 2018b):

Reproductive health is a state of complete physical, mental and social well-being and not merely the absence of disease or infirmity, in all matters relating to the reproductive system and to its functions and processes (United Nations, 2015b).

While these documents are not binding for the countries that sign them, progress has been made in several aspects of sexual and reproductive health and rights since the ICPD conference in 1994. For instance, the rates of maternal mortality and morbidity have improved in all parts of the world (Timothy N Thomas et al., 2014a, 2014b; World Health Organization, 2023). Contraceptive use rates have increased and access to family planning services has improved (Becquet et al., 2024), although sometimes such progress has unfortunately been achieved using coercive measures, which violate human rights and as such sexual and reproductive health and rights as well (Senderowicz, 2019). New technologies have improved the treatment and prevention of sexually transmitted infections, such as HIV, as well as helped many struggling with infertility or subfertility to conceive using assisted reproductive technologies, such as in-vitro fertilization(IVF) (Becquet et al., 2024).

However, progress has not been uniform across all aspects of sexual and reproductive health. According to the Guttmacher-Lancet commission the following aspects remain overlooked in international policy: safe abortion services, treatment of infertility, prevention of cervical cancer, sexually transmitted infections, and violence against women and girls (Becquet et al., 2024). Another recent review of progress since the ICPD conference of 1994 also highlighted several areas of improvement especially in equality of access to advancement in sexual and reproductive health. These include the absence of men from family planning policy documents and actions; the rollback of safe abortion access in many countries; lack of access to assisted reproductive technologies in poorer countries and among less advantaged population groups in high-income countries; new HIV prevention and treatment tools being inaccessible for the poorest populations of the world; and the exclusion of gender and sexual minorities from the Programme of Action document (Becquet et al., 2024).

This commentary explores in more detail many of the issues that have received less international attention, where progress has been slow or unequal, and important global and/or gender disparities remain. We draw on the authors' specialty areas, thus focusing on some, but not all, of the issues mentioned above. The topics discussed in this commentary are introduced in more detail below.

First, we examine the advancements in treatment and prevention of sexually transmitted infections, including but not limited to the human immunodeficiency virus (HIV). We review the decreasing risk of transmission of HIV among those under treatment, and the potential of pre-exposure prophylaxis (PrEP) to protect those at high risk of HIV infection and pre-exposure prophylaxis (Doxy-PEP) to prevent bacterial sexually transmitted infections. We also discuss the revolutionizing nature of human papillomavirus (HPV) vaccination in prevention of reproductive cancers. Finally, we highlight the work that remains by examining inequities in access to treatment, antimicrobial resistance, persisting stigma around these issues, and the changing global health landscape including funder availability and priorities.

Second, we turn our focus on assisted reproductive technologies and changes in the treatment of in/subfertility since the ICPD conference in Cairo. We examine the extent to which environmental issues and health problems can shape one’s fertility, inequalities in accessing assisted reproductive technologies across and within countries, as well as vulnerabilities and violences related to this type of healthcare. We also highlight the future impacts of non-curative use of assisted reproductive technology, such as planned oocyte cryopreservation, and the use of these technologies among same-sex couples and single women.

Third, we review the state of reproductive health among those who experience pregnancies not ending in a live birth, that is in most cases induced abortions, miscarriages, ectopic pregnancies, and/or stillbirths. We highlight the advances, setbacks, risk factors, and potential negative consequences of each pregnancy type depending on the context and circumstances in which the pregnancy is experienced. We stress the social inequalities in these experiences persisting within and across nations. Finally, we discuss the emerging concerns in this domain, notably the effects of climate change, and the rollback of sexual and reproductive rights in many parts of the world. The three topics unfold sequentially along the continuum of human reproduction—from maintaining sexual health, to achieving conception, and finally to carrying a pregnancy to term.

Overall, this commentary aims to highlight at the same time some of the most important improvements in these areas of sexual and reproductive health, as well as discuss setbacks and areas for improvement. We also turn our gaze towards emerging concerns and policy needs in the future.

## HIV and other sexually transmitted infections

### Progress since ICPD

Over the past three decades, remarkable progress has been achieved in the area of HIV and other sexually transmitted infections (STIs) towards the objectives listed in the Programme of Action—Chapter VII “Reproductive Rights and Reproductive Health”, Section C—to focus on the prevention and control of STIs– and Chapter VIII “Health, Morbidity and Mortality”, Section D on HIV/AIDS as a health challenge (UN Department of Economic & Social Affairs, 1994). This progress has been driven in part by novel biomedical tools that became available over the past 30 years. Below, we summarize key aspects where novel technology and strategy were fueled by scientific discovery and have revolutionized the prevention and treatment of HIV and other STIs to safeguard population health.

#### PrEP

The Programme of Action emphasized condom use as the mainstay for HIV/STI prevention of that time (UN Department of Economic & Social Affairs, 1994). The subsequent years witnessed the expansion of biomedical intervention options, which vastly enriched our prevention toolbox against HIV infection, and the initiation of exploring biomedical preventive options for non-HIV STIs. Among the most transformative developments in HIV prevention have been the introduction of PrEP, defined by the World Health Organization as “the use of an antiretroviral medication by HIV-negative people to reduce the risk of HIV acquisition”(World Health Organization). PrEP significantly reduces the risk of HIV acquisition across multiple sub-populations who are disproportionately affected by HIV, including men who have sex with men (MSM), HIV serodiscordant couples, and people who inject drugs (PWID) (Baeten et al., 2012; Choopanya et al., 2013; Grant et al., 2010). The integration of antiretroviral medications for pre-exposure protection has provided a powerful complement to behavioral and structural interventions, such as condom use and community mobilization, both recommended in the Programme of Action (UN Department of Economic & Social Affairs, 1994).

Moreover, a study conducted in rural Kenya and Uganda demonstrated that PrEP uptake among individuals, compared with matched controls, reduced HIV incidence by 76% among women, which is even higher than the average incidence of 74% across both genders (Koss et al., 2021). This suggests that biomedical prevention is practical and feasible among women in generalized-epidemic settings, providing critical protection for women who face heightened risk of and vulnerability to HIV compared to men. The emergence of PrEP-related studies and evidence generation across the Global North and South documented the strengthened global HIV response and collective effort to improve population health across borders in the past three decades since ICPD. PrEP, despite not yet existing during ICPD in Cairo, aligns closely with the ICPD’s rights-based approach to sexual and reproductive health by broadening the range of prevention options for individuals and particularly empowering women.

Efficacious PrEP depends on adherence to the regimen by its users, which poses challenges to certain people (O Murchu et al., 2022). To address this challenge, expanding PrEP modalities, including the option of long-term injectable PrEP, further adapted preventive technologies to meet persons’ needs (Marshall et al., 2018). Taken together, this progress translated the ICPD’s normative commitment into actionable HIV prevention actions to improve sexual and reproductive health of the population at risk.

#### Treatment as prevention

Besides PrEP, HIV treatment coverage has expanded worldwide over the past three decades, driven by evolving guidelines, improved access, global funding commitment, and public health initiatives. The trend of increased coverage of antiretroviral therapies was most pronounced in the past two decades, evident in countries such as Ghana, where ART coverage increased from 1% to 2004 to 60% in 2020 (Boah et al., 2023), and Ethiopia, where coverage nearly doubled from 31% in 2010 to 59% in 2016 (Girum et al., 2018). These outcomes demonstrated the successful fulfillment of the objectives listed in the Programme of Action Chapter VIII, sub-section D “Human immunodeficiency virus (HIV) infection and acquired immunodeficiency syndrome (AIDS)”, which underscored “to ensure that HIV-infected individuals have adequate medical care” (UN Department of Economic & Social Affairs, 1994).

In an era of widespread HIV treatment coverage, the field has moved well beyond ICPD’s action item 8.35, which recommended “voluntary sexual abstinence” as part of “responsible sexual behavior” for HIV prevention (UN Department of Economic & Social Affairs, 1994). Enabled by ART, the development of Treatment as Prevention (TasP) and the Undetectable = Untransmittable (U = U) principle marks a fundamental paradigm shift with broad and lasting implications. The TasP strategy, empirically supported by early cohort studies such as Rakai study in Uganda, and further confirmed by randomized trials such as HPTN 052 in 2011, suggested that consistent and effective ART use lowers the HIV viral load to undetectable levels, reduces infectiousness to other persons at risk, and therefore limits onward sexual transmission of HIV (Grinsztejn et al., 2014). Based on these findings, serodiscordant couples no longer have to rely on sexual abstinence as the sole means to prevent HIV infection. They can now have safe and pleasurable sex, a right enshrined in the definition of sexual health (Glasier et al., 2006). Following TasP discovery, several cohort studies demonstrated consistent results, including the PARTNER 2 study (Rodger et al., 2019) conducted in 14 European countries among serodiscordant gay male couples, and the “Opposites Attract” observational cohort study conducted among similar key population groups from Australia, Brazil, and Thailand (Bavinton et al., 2018). Thereafter, the evidence base has been sufficiently built for policymakers and international organizations to follow up with legislative action and advocacy. Launched and widely endorsed in 2016–2017, the U = U messaging distilled scientific evidence on TasP into a powerful, comprehensible public health campaign that essentially transformed HIV-related social discourse. U = U not only reaffirmed the preventive power of ART but also played a critical role in destigmatizing people living with HIV by emphasizing their active contribution to epidemic control. Furthermore, U = U has the potential to resolve to a certain extent the tension within serodiscordant partnerships, and therefore mitigate the negative impact of HIV on marriage and partnership from a demographic perspective.

In conclusion, TasP and U = U bridge treatment and prevention, reinforcing the principle that health interventions must serve both population-level control of disease transmission and individual well-being. Moreover, they highly align with the ICPD’s human-centered ethos by framing HIV treatment as a pathway to dignity, social inclusion, and collective well-being of dyadic relationships, larger interpersonal networks, and communities, rather than solely as a medical necessity. Even though neither U = U nor TasP were foreseeable in the Programme of Action in 1994, they embodied the ICPD's commitment by exemplifying the continued evolution of the ICPD agenda—where scientific advancement and policy and advocacy converge to achieve progress in HIV prevention and treatment.

#### Non-HIV STIs

Non-HIV STIs received less mention in the Programme of Action compared to HIV. In terms of cervical cancer and HPV, the Programme of Action emphasized “further diagnosis and treatment for … cancers of the reproductive system” as a key action item in 7.6 (UN Department of Economic & Social Affairs, 1994). Scientific breakthroughs had started before ICPD in 1994, gradually establishing evidence regarding the causal effect of HPV on cervical cancer (Schwarz et al., 1985). Following the epidemiological confirmation that HPV types 16 and 18 are causally linked to cervical cancer, HPV has become the focal point of subsequent cervical cancer prevention policies and programs.

These scientific breakthroughs spurred notable changes in clinical and public health practices. Firstly, HPV detection through DNA sequencing has enabled “earlier than early” detection of cervical cancer by identifying the carcinogenic risk factors (Crosbie et al., 2013). Secondly, vaccination against HPV infection provided a novel means for protecting people at risk against the infection of HPV through sexual intercourse (Roden & Stern, 2018). In 2006, the introduction of the first HPV vaccine—and its subsequent global scale-up—significantly redirected cervical cancer control efforts from early diagnosis toward proactive prevention of infection with carcinogenic HPV types. (Herrero et al., 2015). Nowadays, HPV vaccination is widely recommended by the World Health Organization (WHO) Strategic Advisory Group of Experts on Immunization, for girls aged 9–14, before the start of sexual activity and further for, where feasible and affordable, boys and older girls and women (World Health Organization, 2022).

The development and implementation of HPV vaccines fundamentally altered the global landscape of preventing cervical cancer. Since their introduction, national immunization programs in many countries have progressively integrated HPV vaccines for eligible individuals, leading to measurable reductions in HPV prevalence, genital warts, and cervical precancerous lesions (Spayne & Hesketh, 2021). The coverage of HPV vaccination among adolescents, and, in some countries, through school- or community-based delivery models, represents a public health milestone consistent with the ICPD’s emphasis on universal access to preventive services and health equity. Despite persistent gaps in low- and middle-income countries (Bruni et al., 2016), the global momentum toward HPV elimination underscores the translation of the ICPD’s vision into practice—linking sexual health promotion, gender equity, and primary prevention.

Beyond HPV, other STIs have gained increasing attention in both research and programmatic work. One of the notable recent strategies is the use of doxycycline as post-exposure prophylaxis (doxyPEP) to reduce bacterial STIs, particularly among MSM and transgender women. Findings from recent clinical trials and real-world studies led to new guidelines and raised important questions about its effectiveness and potential risks among people at risk of bacterial STIs and re-infection (Bachmann, 2024). DoxyPEP used within 72 h after condomless sex was found to reduce the incidence of chlamydia and syphilis by 65–77% and 70–80%, respectively, in MSM and transgender women, with a more modest effect on gonorrhea (about 12–50%) (Luetkemeyer et al., 2023, 2025; Molina et al., 2018; Sokoll et al., 2025). Real-world data confirm significant reductions in STI rates in these groups (Sankaran et al., 2025; Traeger et al., 2025). However, another experimental study carried out among cisgender women did not confirm DoxyPEP’s effectiveness in lowering overall STI incidence, likely due to high baseline doxycycline resistance in gonorrhea and low adherence (Stewart et al., 2023). To date, this topic remains to be further explored.

Emerging empirical studies on DoxyPEP directly respond to the ICPD Programme of Action’s call for comprehensive STI prevention, early detection, and treatment—particularly at the primary health care level. Doxy-PEP research contributed to the overall trend whereby contemporary STI control strategies move beyond condom-centered behavioral interventions to integrate biomedical innovation. Together, HPV vaccination and Doxy-PEP represent key progress on the STI control agenda outlined in the ICPD Programme of Action, reinforcing its core principles of “accessibility”, “prevention”, and rights-based health care through biomedical scientific advancement (UN Department of Economic & Social Affairs, 1994).

### Emerging challenges

Although these technological advancements offered powerful new tools, their implementation revealed several practical challenges, some of which have persisted for decades. Those challenges can broadly be categorized into increasing antimicrobial resistance, inequitable resource distribution, and persistent stigma.

#### Antimicrobial resistance

In the treatment of non-HIV STIs, antimicrobial resistance (AMR) increasingly threatens the effectiveness of current therapies and increases the risk of untreatable infections, especially for high-risk sexual networks (Jensen & Unemo, 2024). Among common STIs, antimicrobial resistance in *Neisseria gonorrhoeae* has already rendered several antibiotic classes ineffective, and its emerging resistance to the remaining first-line agents demands urgent attention, given the risk of exhausting last-resort treatment options. *Mycoplasma genitalium*, although less commonly reported, also shows a rising trend of resistance to macrolides and quinolones, with one recent study reporting macrolide resistance rates above 70% (Ring et al., 2022). Resistance in *Chlamydia trachomatis* and *Treponema pallidum* (syphilis) is less commonly reported.

Such AMR not only compromises STI management but also has broader implications for sexual and reproductive health, including increased risk of pelvic inflammatory disease, which increases the risk of infertility, and adverse pregnancy outcomes, jeopardizing the gains in reproductive health. Similarly, rising antimicrobial resistance in urinary tract infections, such as multidrug-resistant *Escherichia coli*, affects women’s reproductive health and contributes to treatment failures and complications in the service delivery of sexual and reproductive health care (Stapleton et al., 2020). Addressing AMR requires integrated surveillance, rational antibiotic use, and investment in new treatment regimens—measures consistent with the ICPD’s call for “high-quality” services in sexual and reproductive health (UN Department of Economic & Social Affairs, 1994).

#### Inequity and stigma

Despite substantial gains in prevention science, access to STI and HIV prevention services remains deeply uneven within and across countries. In high-income settings, PrEP uptake is much higher among white men and lowest among ethnic minority women, mediated by access to health insurance(Johnson et al., 2022; Odii et al., 2024). In many low- and middle-income settings, the high cost of PrEP, combined with fragmented procurement systems, continues to limit its uptake and continued use of PrEP among people at greatest risk. In Cameroon, for example, fewer than half of eligible key population members initiated PrEP, and adherence remained low (Ndenkeh et al., 2022). As a result, marginalized groups globally still shoulder a disproportionate share of new infections and tend to be the ones most likely alienated and prevented from benefiting from new HIV/STI preventive technology. These patterns underscore the need to match biomedical innovation with explicit commitments to social justice. Without equitable distribution mechanisms and sustainable financing, the next generation of preventive technologies could entrench—rather than alleviate—existing health inequalities, running counter to the ICPD’s vision of universal access to sexual and reproductive health care.

Stigma and discrimination persist as major obstacles to effective HIV and STI responses, despite decades of activism, policy reform, and public health investment (Ncitakalo et al., 2021). For many key populations, the biological burden of disease is compounded by social marginalization, criminalization, and exposure to violence (Lyons et al., 2023). These structural determinants impede equitable uptake of services. For example, stigma around PrEP use could hinder its uptake (Golub, 2018). The Programme of Action’s section on “Prevention and treatment of sexually transmitted diseases, including HIV/AIDS” called for reducing stigma and violence against people living with HIV and other vulnerable groups, including women and young people. It emphasized that governments have an obligation to ensure that individuals are not denied essential information for preventing transmission and can access treatment and care without fear of discrimination or harm (Chapter IV, p. 246) (UN Department of Economic & Social Affairs, 1994). Although the ICPD Programme of Action did not explicitly name specific key populations, it placed human rights, gender equality, and participatory health governance at the center of efforts to safeguard the health of vulnerable groups. Contemporary rights-based frameworks build on this foundation by treating stigma reduction and community empowerment as core components of effective disease control. Community-based organizations remain essential not only for delivering services but also for shaping social norms and ensuring policy accountability—an enduring expression of the ICPD’s principle that communities are both agents and beneficiaries of development (Yang et al., 2020).

## Infertility

In the ICPD Programme of Action, infertility appears in two main contexts. First, within the framework of sexual and reproductive health services, infertility care is explicitly identified as part of the essential sexual and reproductive health package to be delivered through primary health care and supported by referral systems. This is underscored in the action item 7.6 on “prevention and appropriate treatment of infertility”, which states that “all countries should ensure universal access to reproductive health through primary care by 2015”, including the “prevention and appropriate treatment of infertility” (UN Department of Economic & Social Affairs, 1994). The document further calls for reliable referral pathways for “further diagnosis and treatment for complications of pregnancy, delivery and abortion, *infertility*, reproductive tract infections, breast cancer and cancers of the reproductive system, sexually transmitted diseases, including HIV/AIDS” (UN Department of Economic & Social Affairs, 1994). In the section on national action, governments are also tasked with the “prevention of infertility and appropriate treatment, where feasible” in Basis for Action 13.14 (UN Department of Economic & Social Affairs, 1994). What remains unstated, however, is the degree to which medical intervention in human fertility is envisioned, including which infertility services should be considered essential, ethical, and person-centered. Second, the Programme of Action emphasized infertility as a preventable consequence of untreated sexually transmitted infections. It highlights in Basis for Action 7.28, that, for women, STI symptoms are often less visible and therefore more challenging to diagnose, yet carry more severe long-term consequences, including increased risks of infertility and ectopic pregnancy (UN Department of Economic & Social Affairs, 1994).

Thirty years later, infertility has resurfaced as a far more complex public health and demographic issue, shaped in part by widespread postponement of childbearing among younger generations. Age-related declines in fecundity lengthen time-to-pregnancy, pushing more individuals and couples into the medical category of infertility (Hull et al., 1996). At the same time, research has deepened our understanding of multiple levels of determinants of infertility, highlighting environmental exposures as additional risk factors (Mendola et al., 2008; Vélez et al., 2015). Infertility now must be examined not only through its association with untreated STIs in individuals’ medical history, but also in relation to everyday chemical exposures, to factors linked to modern lifestyle patterns, to factors tied to delayed attempts at parenthood, and to a substantial share of cases with no identifiable causes. The following sections outline the key scientific advances and societal changes since the 1994 ICPD conference in Cairo—both of which have transformed the landscape of infertility—before turning to the persistent and emerging challenges that arise at the intersection of infertility and broader social determinants.

### Changes since ICPD

Infertility is commonly defined as “the failure to achieve pregnancy after 12 months of regular unprotected sexual intercourse.” (Carson & Kallen, 2021) It is a definition anchored in the absence of a particular outcome within a specific timeframe. Because of this, infertility is partly a behavioral and social construct, leaving room for ambiguity in medical interpretation and flexibility in clinical intervention. Over the past three decades, technological progress has transformed treatment options and, in many ways, revolutionized human reproduction. This happened in tandem with demographic changes, i.e. childbearing postponement, rising singlehood, the diversification of partnerships beyond heterosexual unions, and increasing maternal age. Together, these domains have created a wide array of new opportunities and challenges in the prevention and treatment of infertility.

#### Advancements in assisted reproductive technology

Although IVF existed well before the 1994 ICPD in Cairo—the first IVF birth occurred in 1978—the technology did not reach today’s nearly 50% success rates until much later. Since the ICPD, IVF success rates have steadily improved, accompanied by a continuous rise in the annual number of treatment cycles performed worldwide. Access has also expanded: in the context of sustained fertility decline, many governments have adopted more proactive policies, subsidizing treatments such as ovulation induction, IVF, and related interventions (Hoorens et al., 2007).

The introduction of intracytoplasmic sperm injection(ICSI) in 1992 represented a major breakthrough, enabling fertilization using a single sperm and addressing severe male-factor infertility (ESHRE Capri Workshop Group, 2007). Cryopreservation techniques have also advanced significantly. Modern vitrification can preserve oocytes with minimal cellular damage, effectively extending reproductive potential for women (Fahy et al., 1984). As success rates climbed, clinical guidelines shifted away from the earlier reliance on transferring multiple embryos (Practice Committee of the American Society for Reproductive Medicine & the Practice Committee for the Society for Assisted Reproductive Technologies, 2021). The recent proliferation of embryo vitrification and genetic screening illustrates a rapid maturation of assisted reproductive technology. These available techniques intersect directly with changing fertility behaviors and together carry important demographic and social implications (Inhorn & Birenbaum-Carmeli, 2008). Today, an estimated 5% of births result from IVF in countries that provide strong public subsidies; IVF has notably contributed to the rising share of births among women aged 35 and older, and even 40 and above (Lazzari et al., 2021).

#### Emerging demographic phenomena

Over the last 30 years, rising educational attainment and expanded female labor-force participation have contributed to widespread fertility postponement and declining birth rates. Fecundity—the biological capacity to reproduce—declines with age in both sexes, with the decline occurring earlier and more sharply in women (Sauer, 2015; Wesselink et al., 2017). This pattern reflects diminishing gamete quality and, to a lesser degree, age-related changes in the reproductive tract. Technological innovation, particularly oocyte vitrification, has enabled elective or “social” oocyte cryopreservation, allowing women to store reproductive potential for future use.

Family structures have diversified as well. Female-headed households and single motherhood via donor sperm can be seen as the exercise of women’s reproductive autonomy (Zadeh et al., 2016). Increasing numbers of sexual minority individuals and unmarried women are turning to assisted reproductive technology to realize parenthood, reflecting broader acceptance of diverse family forms and the loosening of the historical link between parenthood, heterosexuality, and marriage (Smietana et al., 2018). Fertility treatment, once designed to address clinically defined infertility, now operates in a much broader social landscape. Elective fertility preservation, assisted reproductive technology use by single individuals and non-heterosexual couples in wedlock, and the integration of genomic screening all signal a redefinition of reproductive medicine’s boundaries. These developments highlight the intersection of fertility postponement, gender equity, and reproductive autonomy—core themes within the ICPD’s human-centered framework.

The ICPD Programme of Action proposed principles but lacked guidance on the role of infertility treatment, including reproductive technologies, and on the governance structures required to manage them. As a result, countries today maintain widely divergent legal frameworks regulating infertility treatment and prevention. Despite substantial technological maturation, clinical practice and moral gatekeeping still exist, which may limit access for certain groups. These tensions reflect urgent contemporary challenges and underscore the need to balance technological development and the renewed commitment to social justice—ensuring that expanding reproductive choices do not exacerbate existing gender and socioeconomic inequalities.

### Challenges in intersectional areas

#### Uneven access and utilization

Access to infertility diagnosis and treatment remains starkly stratified, both within and across countries. High-income countries have seen rapid expansion of fertility treatment facilities, insurance coverage, and technological sophistication, but also disparity in access to infertility treatment by racially and economically disadvantaged subgroups (Ekechi, 2021). In contrast, many low- and middle-income countries (LMICs) lack even basic diagnostic capacity or affordable treatment options (Nachtigall, 2006). The cost of a single IVF cycle can exceed annual household income in most low-resource contexts, excluding the majority of those affected.

Access to infertility treatment services is severely limited in many sub-Saharan African countries. IVF and related medical services remain scarce due to high costs, limited public funding, insufficient infrastructure, and sociocultural barriers (Whittaker et al., 2024). This widens the treatment gap between the Global North and the Global South. Historically, reproductive health funding—especially in LMICs—has prioritized family planning and contraception, mirroring demographic and policy agendas to reduce population size, but systematically sidelining women’s unmet need for infertility care (Mutumba Nakalembe & Janes, 2025). Since the ICPD in Cairo until now, global reproductive health discourse has centered on fertility limitation, overshadowing the burden of infertility in sub-Saharan Africa; today, availability and coverage of infertility services in LMICs remain largely stagnant. This inequity contradicts the ICPD principle of universal access to reproductive health services, of which infertility treatment represents an integral component. Infertility, recognized by the WHO as a disease of the reproductive system, has recently seen its first published WHO official treatment guideline (Mburu et al., 2025). Closing the affordability and availability gap will also require context-appropriate, low-cost fertility treatment methods and the integration of infertility services into universal health coverage benefits.

By contrast, some high-income settings face over-utilization of medically assisted reproduction. Sociologists argued that commercial promotion of assisted reproductive technology, such as oocyte cryopreservation, can obscure the structural constraints—insufficient workplace support, gender discrimination, precarious employment—that shape women’s reproductive timing (Myers & Martin, 2021). Furthermore, the proliferation of fertility treatment “add-ons,” many lacking robust evidence of benefit, further complicates clinical decision-making and makes it harder for individuals to make choices as they navigate the complex treatment arena loaded with medical jargon (Galiano et al., 2021; Huang, 2022). Even within high-income countries, disparities occur where subsidization is insufficient: highly educated women are consistently more likely to access and benefit from infertility treatment in the United States (Goisis et al., 2024).

#### Intimate partner violence in the context of infertility

Beyond structural inequities, intimate partner violence (IPV) and coercion are increasingly documented among individuals with infertility. Women undergoing infertility treatment may face psychological, sexual, or economic abuse from partners or extended family (Fu et al., 2024). A systematic review study reported prevalent IPV against infertile women in LMICs (Wang et al., 2022). These consequences arise from the intersection of infertility and gender inequality—two themes emphasized in the Programme of Action—and increasingly warrant scholarly attention and policy responses.

#### Environmental exposures

The epidemiology of infertility is evolving alongside broader development in environmental and population health research. For example, researchers observed that environmental factors may exacerbate the risk of infertility by amplifying the effects of pre-existing genetic risk factors (Oliva et al., 2001). In recent years, various studies investigated the exposure to endocrine-disrupting chemicals, air pollution, and heavy metals and their potential linkages to infertility through mechanisms such as impairing male sperm quality, altering female ovarian function, and increasing the risk of pregnancy loss (Skakkebæk et al., 2022). Among males (Min & Min, 2017; Petrelli & Mantovani, 2002), exposure to pesticides and noise was examined in relation to fertility, while among females, psychological stress and substance consumption (such as alcohol, tobacco, and caffeine) were scrutinized as potential risk factors (Bala et al., 2021). These environmental factors interact with lifestyle-related risks—obesity, delayed childbearing, sexually transmitted infections—to create a complex web of biological and social determinants. Future studies are warranted to fully tease out the potential contributions of social, economic, and behavioral determinants, as well as environmental exposure, both of which correlate with industrialization and modern lifestyles. From an ICPD perspective, these patterns emphasize the importance of integrating infertility prevention into broader public health, environmental health, and sustainable development agendas.

### A call for an ethical framework for infertility treatment and prevention

The ICPD’s commitment to reproductive autonomy and access to infertility-related services provides a strong base for regulating emerging reproductive technologies. Building on that foundation, maintaining ethical standards, ensuring responsible use of fertility treatments, requiring informed consent, and addressing stigma and violence linked to infertility are crucial—not only to protect individuals but also to align infertility care with broader reproductive justice principles. Besides, the uneven global diffusion of fertility technologies highlights a stark dual challenge: severe shortages of essential infertility services in the Global South and, conversely, the risks of over-medicalization and inequitable access in the Global North. The rapid globalization of the fertility industry underscores the need for a robust ethical and regulatory framework that can keep pace with scientific innovation. At the same time, preventing the excessive commodification of fertility care is critical to avoiding new forms of vulnerability and exploitation. The ICPD’s emphasis on dignity, autonomy, and well-being remains profoundly relevant. The next phase of the global reproductive health agenda must reconcile technological progress with social justice—ensuring that assisted reproduction becomes a mechanism for empowerment rather than a source of exclusion.

## Pregnancies not ending in live births

While advances have been made since the ICPD conference in Cairo in 1994 in maternal health (Thomas et al., 2014a, 2014b), the process has been more complicated for pregnancies not ending in live births. Such pregnancies include spontaneous pregnancy losses (most commonly miscarriages, ectopic pregnancies, and stillbirths) and induced abortions. None of these issues were priorities in the Programme of Action published after the ICPD conference (UN Department of Economic & Social Affairs, 1994), most likely due to political tensions, lack of research and understanding of these issues, and various stigmas and taboos, which are linked to these pregnancy outcomes in most contexts. Below, we discuss the specific issues related to these pregnancy outcomes, inequities associated with these experiences, and emerging concerns that the academic and medical communities should monitor in the years to come.

### Specific issues related to pregnancies not ending in a live birth

#### Induced abortion

Induced abortions are intentional terminations of pregnancy either by a medical professional, the pregnant person, or another third party, using medication, a chirurgical operation, or other means to end the pregnancy. When conducted using safe methods and by trained professionals, induced abortion is one of the safest medical procedures (Kapp & Lohr, 2020), whereas unsafe abortions can result in important health complications or even death of the pregnant person. Around eight percent of maternal deaths annually are estimated to be due to unsafe abortion, the vast majority taking place in low-income countries (Say et al., 2014).

As already discussed elsewhere (Becquet et al., 2024), a right to induced abortion was not mentioned in the Programme of Action document due to opposition from more conservative delegates, but the necessity of access to post abortion care was mentioned. Since the ICPD conference, many countries in particular in Latin America, have liberalized their abortion access, but other countries have implemented important restrictions, including Poland and the United States (Becquet et al., 2024). Given the wide-ranging political and economic influence of the United States in the world, the attacks on sexual and reproductive health and rights, including the right to abortion, are a cause for concern. Previous studies have already demonstrated the devastating health and economic impact of abortion restrictions (Barot, 2011). While the reasoning for such restrictions is often articulated as being due to a desire to reduce the number of abortions, we know from academic literature that abortions happen regardless of their legal status (Bearak et al., 2020), it just makes abortions more difficult to obtain especially for those lacking the economic or social resources to procure a safe abortion in a restrictive environment. While self-managed medication abortion has made clandestine pregnancy terminations much safer (Berro Pizzarossa & Nandagiri, 2021), it is not the appropriate or preferred method in all cases, and abortion restrictions also put in danger pregnant people who are not seeking abortions, as discussed in more detail below. Access to safe and free abortion services is a crucial part of sexual and reproductive health and rights principles (Starrs et al., 2018a, 2018b), as well as a necessary condition for reproductive justice, which advocates the right for everyone to choose if and when to have children and to raise them in safe conditions (Luna & Luker, 2013; Ross, 2020).

#### Miscarriage and other early pregnancy losses

Miscarriages are spontaneous early pregnancy losses (where early can mean anything until between 20 and 28 gestational weeks, depending on country and time period (Prior et al., 2017), which are estimated to affect around 15–25% pregnancies and one in four women/people with uteruses by the end of their reproductive life span (Delabaere et al., 2014; Quenby et al., 2021; San Lazaro Campillo et al., 2019). Ectopic pregnancies mean that an embryo has implanted outside the uterus, often in a fallopian tube, and there is no possibility for such pregnancy to be evolutive.

Miscarriages, ectopic pregnancies, or other similar reproductive complications, such as molar pregnancies or blighted ova, were not specifically mentioned in the Programme of Action document. However, the document mentioned rights and recommended actions, which could be interpreted to be beneficial for the treatment of these types of pregnancies. These include, for instance, “the right of access to appropriate health-care services that will enable women to go safely through pregnancy” and the availability of “diagnosis and treatment for complications of pregnancy” (UN Department of Economic & Social Affairs, 1994).

While some miscarriages require no medical intervention, some can pose a risk to the health or even life of the pregnant person (Say et al., 2014). Ectopic pregnancies often need urgent care to avoid life-threatening complications. While few decision-makers or politicians are openly opposed to this type of healthcare, ample evidence shows that restrictions on abortion access make miscarriages and ectopic pregnancies more dangerous for the pregnant person (Nandi et al., 2024; Stein et al., 2023). For example, rates of hemorrhage have increased as a result of abortion restrictions in the US due to the medical doctors being worried about legal consequences of treating patients, if fetal cardiac activity can still be detected, even if the pregnancy is already known to be non-evolutive or dangerous to the health and/or life of the pregnant person (Nandi et al., 2024).

While abortions and spontaneous early pregnancy losses are often treated as separate issues, there is considerable overlap between them, and any erosion in access to safe healthcare for one issue will affect the other as well.

#### Stillbirth

Stillbirths are spontaneous pregnancy losses late in the pregnancy, where the definition of late varies depending on the country and time period, between 20 and 28 gestational weeks, and some definitions include an additional criterion of the weight of the fetus being at least 500 g (Kelly et al., 2021; Taviani et al., 2024). Like miscarriages, stillbirths were not explicitly mentioned in the Programme of Action, but the general aspirations for a healthy pregnancy and childbirth mentioned in the document, could, if broadly implemented, be beneficial in stillbirth prevention as well. While stillbirths are relatively rare, estimated around 1% of births in high-income countries (HICs) and 3% in LMICs (Say et al., 2006), they are often associated with devastating mental health and other negative consequences (Heazell et al., 2016). The risk of stillbirth might increase due to poor access to healthcare services during pregnancy, and in case of restrictions in access to abortion due to medical indications and/or during the second trimester, due to more pregnancies known not to be viable outside the womb being carried to term. Stillbirths are affected by abortion restrictions and by the general erosion of sexual and reproductive health and rights, and should not be discussed in isolation from other pregnancy outcomes.

### Social inequalities in pregnancy experiences and their consequences

Social inequalities in sexual and reproductive health and rights were not in an important role in the original ICPD declaration, which was a relatively short document. There are a few dimensions of inequality mentioned, though, including concerns about ‘marginalized communities’ and gender inequalities in a few places, and an entire sub-section is dedicated to adolescents (UN Department of Economic & Social Affairs, 1994). While these are important dimensions of social inequalities, they alone are not enough to understand or tackle such issues.

The likelihood of experiencing different pregnancy outcomes varies importantly by age, socioeconomic characteristics, race/ethnicity, and different combinations of these characteristics. While some differences are due to personal preferences, for instance, people waiting to be in a certain economic situation and age before having children, other differences are more concerning. For instance, stillbirth rates are higher among more economically disadvantaged population sub-groups as well as racial/ethnic minorities (Rowland Hogue & Silver, 2011; Smith et al., 2025).

Any pregnancy outcome can have potential detrimental consequences on the health and well-being of the pregnant person, if something goes wrong, as discussed above. The likelihood of such adverse outcomes is not independent from the socio-demographic characteristics of the pregnant person. For instance, unsafe abortion poses the most severe risks among poor women in poorer countries, similarly to the dangers associated with giving birth (Say et al., 2014).

Understanding how and why socio-demographic characteristics affect the inequalities in the risk and consequences of adverse pregnancy events is key to finding suitable policy solutions. Any one socio-demographic or economic characteristic alone cannot explain or help tackle inequalities in the likelihood of experiencing different pregnancy outcomes and their possibly negative consequences. Thus, an intersectional lens is needed to understand how inequalities accumulate and multiply at cross-sections of several marginalized characteristics (Kapilashrami, 2019). Coupled with any such analysis, should be the understanding of the structural nature of such inequities (Kapilashrami, 2019). Focusing on interventions aimed at changing the behaviors of individuals without addressing problems at the structural level will not address or permanently change much about inequalities in the risk and consequences of adverse consequences of pregnancies, no matter the specific outcome. Thus, an effective intervention should stem from a careful analysis of the macro-, meso- and micro-environment in which pregnancies take place, including international and national context, local realities, and the characteristics and networks of the individual.

In order for any policy action on dangers and inequalities of pregnancy experiences to be effective, all pregnancy outcomes need to be discussed and brought into the remit of said policies. In many contexts induced abortion is a taboo subject, which cannot be discussed or addressed, even though we know that safe and affordable access to abortion saves lives and preserves the health of the most vulnerable by protecting those seeking abortions as well as those undergoing other types of pregnancy endings, such as miscarriage or ectopic pregnancy (Nandi et al., 2024; Stein et al., 2023). Yet, abortion and the policies surrounding it, are intertwined with other pregnancy experiences, which cannot be treated in isolation.

Finally, the recommended baseline guidance, which should be written into sexual and reproductive health policies is *reproductive justice*, which stresses that the ability to decide if and when to have children is equally important to the possibility to raise one’s children in a safe environment (Luna & Luker, 2013; Ross, 2020). This principle forces one to take into account both the structural and the more micro-level aspects of inequalities when designing policies.

### Emerging concerns

Taking together the issues discussed above, and combining all that with some likely future developments, there are some emerging concerns that are important for pregnancies not ending in a live birth.

First, climate change will increase the likelihood of heatwaves globally in addition to other adverse events, such as storms. Given that heat is a risk factor for miscarriage (Das et al., 2023; Moodley et al., 2024), the likelihood of such events will also increase. In addition, some currently inhabited parts of the globe may become uninhabitable due to the impacts of climate change, which will increase the number of displaced people, who are known to suffer from poor sexual and reproductive health (Logie et al., 2024).

Second, while some countries have made progress in their sexual and reproductive health and rights, many have gone backwards in recent years, most importantly the United States. This includes the supreme court decision, which overturned the federal right to abortion until the point of fetal viability (Dobbs, 2022), as well as the return of the harmful ‘Mexico City Policy’ to any foreign aid programs prohibiting funding to any organization that performs or even informs people of induced abortion (Keith, 2025), until almost the entire USAID budget was cut (Mbah et al., 2025). All these actions will have profound negative consequences to the health and well-being of large numbers of people, including those experiencing pregnancies, no matter their outcome, due to programs supporting safer sexual and reproductive health services being cut.

When publicly discussing climate change or the aforementioned policy and legal changes, it is important to highlight their impact on sexual and reproductive health and rights broadly, including different pregnancy experiences and their consequences. While it is difficult to tackle these issues, which require global co-operation at a time when the political climate is making such co-operation more and more challenging, it is the only route to profound and lasting improvements in sexual and reproductive health and rights instead of their deterioration. It was demonstrated above that different pregnancy experiences are interlinked and improvements for one outcome cannot be made unless the others are also taken into account. Similarly, many of the causes of global and local inequalities in the likelihood of experiencing adverse outcomes due to a pregnancy are and increasingly will be associated with global phenomena, such as climate change and global economic inequities, and thus cannot be tackled by only designing interventions aiming to change behaviors at the individual level. Future monitoring of pregnancies, their outcomes, and associated adverse outcomes is important. This is likely to be difficult, if people do not feel safe disclosing all of their pregnancy experiences, including induced abortions which are already underreported in surveys (Lindberg et al., 2020), but even more important, so that better policies can be designed and put in place. All this needs resources, which should be taken into account by any government hoping to make advances in the field of decreasing inequities in sexual and reproductive health and rights.

## Concluding reflections

Thirty years after the ICPD in Cairo, the global landscape has shifted considerably. A growing number of countries are experiencing a resurgence of conservative values, with significant implications for sexual and reproductive health. These shifts potentially carry two major risks. The first concerns the erosion of evidence-based policy. Conservative ideological movements have led, in some contexts, to restrictions on reproductive health services such as safe abortion. The surge of pronatalist rhetoric may further undermine the individual-centered, rights-based principles championed in Cairo three decades ago. In fact, these trends directly contradict the ICPD Programme of Action, which explicitly states that “*demographic objectives should not translate into client quotas or coercive targets for family-planning providers*” (UN Department of Economic & Social Affairs, 1994). The second risk lies in the reduction in resources allocated for sexual and reproductive health, both internationally and domestically. Studies have shown that reductions in international funding for sexual and reproductive health have generated significant disruptions such as service reductions and clinic closures (Ushie et al., 2020; Vernaelde, 2022). For instance, the recent withdrawal of major bilateral donors is estimated to cause disruptions in sexual and reproductive health programs, forcing recipient countries to reorganize their long-standing HIV/STI response system within a short timeframe in order to avoid severe setbacks (Hussein & Samet, 2025; Schmallenbach et al., 2025). Domestically, unstable or insufficient funding shifts financial responsibility onto individuals, leading to higher out-of-pocket expenditures and widening socioeconomic disparities in access to sexual and reproductive health services in both high-income countries and LMICs (Akazili et al., 2020; Fuentes et al., 2023). For the poorest and most vulnerable groups, even modest increases in cost can translate into delayed care, foregone contraception, and heightened risk of unintended pregnancy or untreated STIs. Moreover, reduced resources threaten the sustainability of local organizations, constrain the generation of new evidence, and weaken accountability mechanisms (Mallik et al., 2023). These setbacks undermine global efforts to advance gender equality, reduce maternal mortality, and achieve universal access to SRH services as outlined in the ICPD Programme of Action.

At this crossroads of uncertainty, nations should take responsibility to ensure the continuity of financing and maintenance of community-based infrastructure that is essential to preserving the current trajectory toward global STI and HIV control (Mulenga et al., 2025). Otherwise, significant adverse impact will occur on sexual and reproductive health services, access, and outcomes, especially for vulnerable populations. The ICPD framework emphasized intersectoral collaboration and long-term institutional investment—principles that remain relevant now, and crucial for safeguarding the gains of the past three decades.

## Data Availability

Not applicable.
